# Policy models for preventative interventions in cardiometabolic diseases: a systematic review

**DOI:** 10.1186/s12913-025-12781-y

**Published:** 2025-05-02

**Authors:** Septiara Putri, Giorgio Ciminata, Jim Lewsey, Hanin Farhana Binti Kamaruzaman, Yuejiao Duan, Claudia Geue

**Affiliations:** 1 Health Economics and Health Technology Assessment (HEHTA), University of Glasgow, Glasgow, UK; 2 Health Policy and Administration Department, Faculty of Public Health, University of Indonesia, Depok, Indonesia; 3 Malaysian Health Technology Assessment Section (MaHTAS), Ministry of Health Malaysia, Putrajaya, Malaysia

**Keywords:** Systematic review, Policy model, Decision model, Cardiometabolic disease, Health economics

## Abstract

**Background:**

Cardiometabolic diseases (CMDs), including cardiovascular disease (CVD) and type 2 diabetes (T2DM), are major contributors to morbidity, mortality, and rising healthcare costs. Effective disease prevention programs rely on robust mathematical models to generate long-term evidence regarding the effectiveness, cost-effectiveness, and policy implications of interventions in the population. Population-level interventions, such as dietary policies, are recognised as essential prevention strategies, yet there is limited syntheis of policy models assessing their impact. This study systematically reviews existing CMD policy models to provide: (i) a comprehensive overview of current models, and (ii) a critical appraisal of their application, particularly in the context of primordial prevention programmes.

**Methods:**

A systematic search was conducted across MEDLINE (Ovid), EMBASE (Ovid), CINAHL, Google Scholar, and Open Grey. The search focused on publications from 1st January 2000, to 31st May 2024, using Medical Subject Headings (MeSH) for “cardiovascular,” “diabetes,” “decision model,” and “policy model.” Full-text articles were independently appraised independently by three reviewers using the Phillips et al. checklist, and the review process adhered to PRISMA guidelines.

**Results:**

Thirty-two articles met the inclusion criteria and were critically appraised. Policy models were assessed across three domains: structure, data, and consistency. Most models (79%) demonstrated well-defined structures, aligning inputs and objectives with the stated perspective and initial justifications. However, fewer than 60% of studies clearly reported the quality of their data sources and provided clear information in terms of consistency. The reviewed studies employed diverse methodologies, including parameter incorporation, simulation modelling, and outcome analysis.

**Conclusion:**

The review highlights substantial heterogeneity in the quality, structure, and data use of policy models evaluating dietary interventions for CMD prevention. To advance CMD policy modeling, this study provides recommendations for improving conceptualisation, methodological rigor, and applicability to prevention programmes.

**Trial registration:**

Registered protocol at PROSPERO: CRD42022354399.

**Supplementary Information:**

The online version contains supplementary material available at 10.1186/s12913-025-12781-y.

## Introduction

Cardiometabolic diseases (CMDs) are a leading cause of disability and mortality, as well as contributing to rising healthcare costs worldwide [1, 2]. CMDs refer to a group of interconnected conditions that include metabolic disorders like type 2 diabetes mellitus (T2DM) and obesity, as well as cardiovascular diseases (CVDs), like heart attacks and strokes, all of which are driven by shared underlying mechanisms such as insulin resistance, chronic inflammation, and dyslipidaemia [3, 4].

The World Health Organization (WHO) reported that diabetes mellitus directly contributed to over 2 million deaths in 2022, with T2DM representing over 90% of cases, while CVDs caused 17.9 million deaths in 2019, accounting for 32% of global mortality [5, 6]. The prevalence of both, T2DM and CVD, is expected to rise significantly over the next two decades. This growing burden of CMDs is driven by a combination of interrelated risk factors, including elevated cholesterol levels, high body mass index, elevated blood glucose, and hypertension. These risk factors are further exacerbated by unhealthy lifestyle behaviours as well as non-modifiable factors like age, race/ethnicity, and family history [5–7]. Given the progressive nature of these diseases and their complex risk profiles, generating robust evidence to inform effective prevention policies remains a significant and important challenge [8, 9].

To evaluate the effectiveness of health interventions, programs, or policies, evidence from randomised controlled trials (RCTs) is often used to support decision-making. However, RCTs face inherent limitations, including high resource and time demands, challenges in generalisability, and their tendency to provide short-term evidence, which can constrain policymakers [10–12]. The chronic and multifaceted nature of CMDs characterised by competing risks, complications, and long-term morbidity, demands evidence that extends beyond what clinical trials typically offer. To address these gaps, modelling is essential for generating long-term projections of intervention outcomes, particularly regarding their effectiveness, cost-effectiveness, and potential implications for health policy. Applying modelling approaches has proven beneficial to assist decision-making processes in public health and policies at various levels [13, 14].

A ‘policy model’ is a structured analytical framework or simulation tool designed to evaluate the potential impacts of policies, programmes, or interventions on population health and healthcare systems over time [10]. These models integrate disease progression, risk factors, economic evaluations, and policy interventions to inform decision-making [13, 15, 16]. In the context of CMD prevention, policy models play a crucial role in assessing primordial or early prevention strategies—interventions that aim to eliminate risk factors before they develop, thereby addressing the root causes of disease at the population level [17]. These include dietary policies, such as sugar taxes, pack labelling, and food reformulation, which are designed to create healthier environments and reduce CMD risk before metabolic disturbance occur.

Several reviews have examined policy and decision models developed for CVD and T2DM, primarily evaluating clinical interventions and cost-effectiveness outcomes [15, 18–27]. However, these models are typically limited in scope focusing on clinical settings, high-risk populations, pharmacological or specific treatment choice, rather than population-wide dietary interventions. Furthermore, several existing models emphasise cost-effectiveness results rather than comprehensive appraisals of the models themselves [20, 23–25].

This, therefore, creates an opportunity to summarise the policy models of two main CMDs such as T2DM and/or CVD. Policy models can be defined as systematic, broad, and comprehensive frameworks explicitly designed to guide and inform policy decisions that impact population-wide health outcomes. Our review will focus on the modelling part, adding more granular information to enrich the appraisal of policy models, particularly modelling for prevention strategies.

This study aims to systematically review the published literature on CMD policy models, with a particular focus on (i) providing a comprehensive overview of existing CMD policy models and (ii) critically appraising their structure and application for primordial prevention programmes.

## Methods

The preferred reporting items for systematic reviews and meta-analyses (PRISMA) guidelines were followed [28]. The review is registered in PROSPERO with registration number CRD42022354399 [29].

### Eligibility criteria

A policy model, as defined in this systematic review, refers to any mathematical, simulation, or framework-based model designed to predict health outcomes, costs, and cost-effectiveness. Given our focus on long-term prevention strategies at the population level, we applied specific inclusion and exclusion criteria to ensure that only relevant models were considered.

We included models that start with a general or low-risk population (i.e., those without clinically diagnosed CMD) to assess the impact of primordial prevention strategies before disease onset. We also required models to predict long-term or lifetime outcomes (≥ 10 years) since policy interventions often have delayed effects on population health. Furthermore, we excluded models focusing on specific subgroups (e.g., obese adults or hypertensive individuals) and those assessing primary prevention with medication, as our interest lies in regulatory and public health measures rather than clinical interventions. The full inclusion and exclusion criteria are detailed in Table 1.

**Table 1:** Inclusion and exclusion criteria

Inclusion	Exclusion
• Models starting with a general or low-risk population and without any CMDs (without clinically diagnoses of CVD/T2DM—disease free) • The model predicts the long-term/lifetime outcomes (> 10 years) • Adult population (≥ 18 years) • Mathematical models that are able to accommodate both health and economic outcomes (cost-effectiveness evidence) • Only models which were assessing and evaluating primordial prevention strategies (restricted to regulations/policy for population dietary, limited to sugar/salt/sodium and fruit/vegetables public health policies) targeting the whole population or population-based prevention	• Clinical studies, cell and animal studies • Models starting with CMD and those for specific subgroups only (such as: obese adults, people with hypertension) • Models focussing on accuracy or cost-effectiveness of diagnostic tools, primary prevention with medication (i.e.: statin use) • Models that reported effectiveness only • Models that were published as presentations, abstracts, commentaries, letters, and review articles

### Search strategy and study selection

A systematic search strategy was developed across multiple databases, including MEDLINE (Ovid), EMBASE (Ovid), CINAHL, Google Scholar, and Open Grey, with publication years restricted to the period between 1 st January 2000 and 31 st May 2024. To ensure a comprehensive review of policy models, we included both peer-reviewed journal articles and relevant grey literature. This approach enhances the breadth of evidence by incorporating real-world policy applications and non-traditional sources. To maintain consistency and accessibility, we limited the review to English-language publications. The search strategy, incorporating Medical Subject Headings (MeSH), is detailed in Supplementary material 1.

To minimise the risk of excluding relevant articles, reference lists of previous systematic and literature reviews were hand-searched using the snowballing technique [30]. The search strategy was collaboratively developed with the support of a University of Glasgow subject librarian and three co-authors (CG, GC, JL). Article management and the removal of duplicates were conducted using Zotero®

### Data extraction

Data from eligible studies were extracted using a standardised matrix in Microsoft Excel®. The extracted items included: author/model name, year of publication, country, model type and structure, perspective, events, outcomes (both clinical and economic), data sources, time horizon, validity, and sensitivity analysis. Data extraction was conducted by a primary reviewer (SP), with independent double-checking performed for 20% of the included studies [31] by co-authors (CG, GC, JL). Discrepancies were resolved through team discussions. Furthermore, to identify and summarise key features from included studies, we synthesised model characteristics by integrating extracted data with a contextual descriptive interpretation of their application. In addition, we also systematically recorded whether and how the included studies reported key aspects such as modelling choices, parameterisation, outcome measures (both health and economic), model validation (e.g., internal and external validation), and sensitivity analysis methods (e.g., deterministic and probabilistic approaches).

### Quality assessment

The quality of reporting for policy models and economic evaluation studies was assessed using the Phillips et al. checklist [16]. This evaluation was conducted by three independent reviewers (SP, HF, YD), with disagreements resolved by consulting co-authors (CG, GC, JL). The results of the quality assessment are presented in a checklist table and illustrated both visually and narratively to provide a comprehensive summary.

## Results

### Selection process

**Fig. 1:**
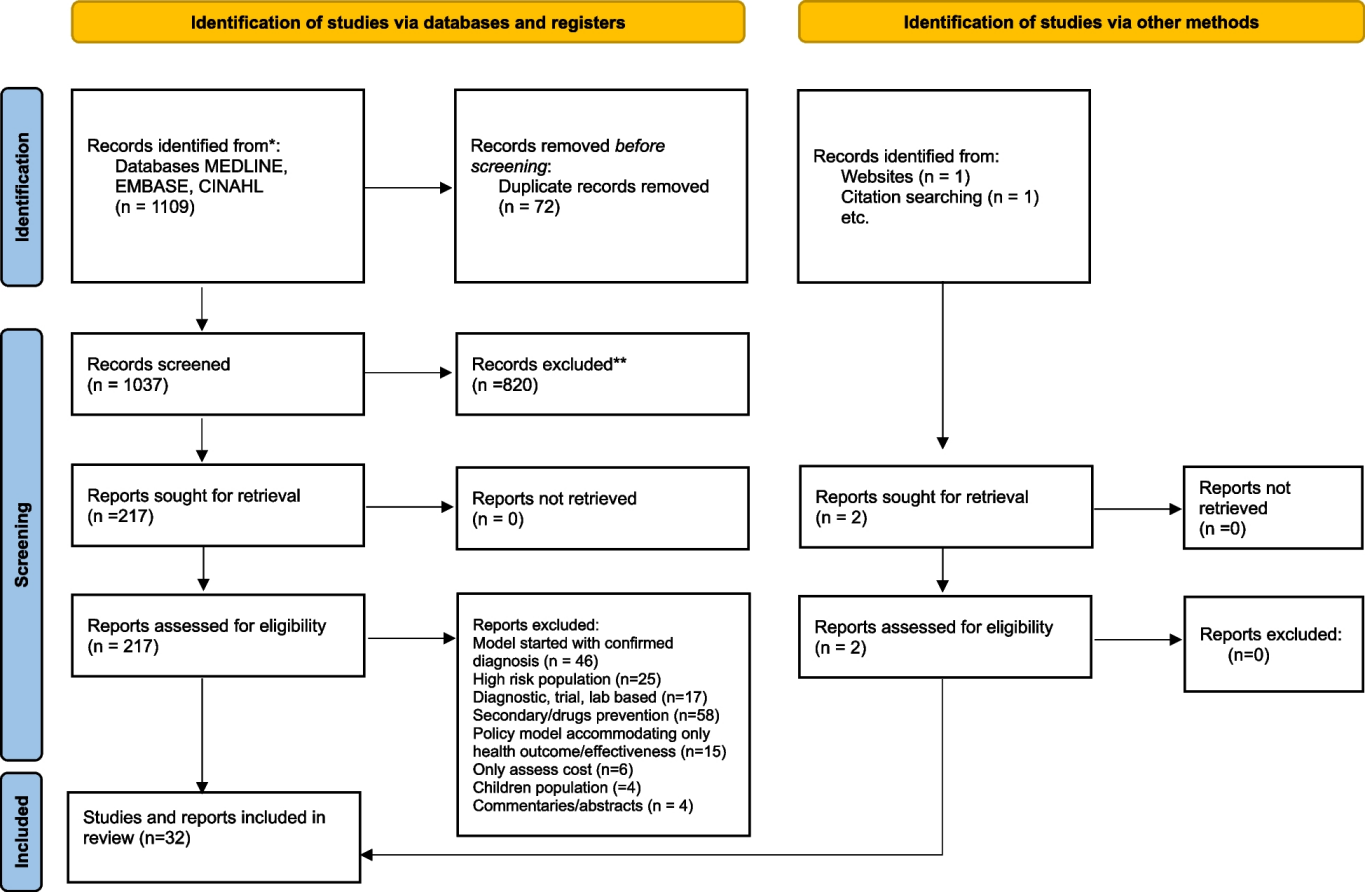
PRISMA 2020 flow diagram

The PRISMA flow diagram (Fig. 1) illustrates the article selection process. An initial search yielded 1109 records, which were reduced to 217 following the removal of duplicates and screening of titles and abstracts. After thorough full-text assessment of these 217 articles, 32 studies met the established inclusion criteria. A summary of these included articles is provided in Table 2.

**Table 2:** Summary of articles included

No	Author (year)	Country	Model’s name	Policy assessment/scenarios/evaluation	Perspective	Model type	Simulation level	Time horizon, cycle	Disease states/measurement
1	Moran et al. (2008) [32]	China	CHD Policy Model-China	Estimation and assessment of the CHD events based on to demographic changes	N/A	Markov	Cohort	30 years, annual	Free CHD, CHD, CHD death, non-CHD death
2	Moran et al. (2010) [33]	China	CHD Policy Model-China	Estimation of future risk factors on CHD and stroke	N/A	Markov	Cohort	20 years, annual	Free CHD, person with CHD, CHD death, non-CHD death
3	Bibbins-Domingo et al. (2010) [34]	US	CHD Policy Model	Estimation of benefits (rates, costs and cost-effectiveness) of salt reduction intervention	Healthcare	Markov	Cohort	10 years, annual	Free CHD, person with CHD, CHD death, non-CHD death
4	Wang et al. (2012) [35]	US	CHD Policy Model	Estimation of potential health impact and spending of a penny-per-ounce excise nationwide tax policy	Healthcare	Markov	Cohort	10 years, annual	Free CHD, person with CHD, CHD death, non-CHD death
5	Basu et al. (2013) [36]	US	-	Estimation of health effects and cost-effectiveness SNAP programme	Government	Microsimulation	Individual	10 years, annual	CVD mortality
6	Konfino et al. (2013) [37]	Argentina	CVD Policy Model-Argentina	Assessment of the impact of sodium reduction policies	N/A	Markov	Cohort	10 years, annual	Free CHD, person with CHD, CHD death, non-CHD death
7	Basu et al. (2014) [38]	India	-	Estimation of the health effect on SSB taxation policy	Government	Microsimulation	Individual	10 years, annual	T2DM incidence
8	Collins et al. (2014) [39]	England	CHD IMPACT Model	Cost-effectiveness analysis of four population health policies on salt intake	Health sector	Cell-based model	Cohort	N/R	CHD death
9	Mason et al. (2014) [40]	Tunisia, Syria, Palestine, Turkey	CHD IMPACT Model	Cost-effectiveness analysis of population-based salt reduction policies in four Eastern Mediterranean countries	Public/private sector, healthcare	Cell-based model	Cohort	N/R	CHD death
10	Lewsey et al. (2015) [41]	Scotland	Scottish CVD Policy Model	The development of CVD policy model that predicts life expectancy and incorporating socioeconomic deprivation	-	Markov	Cohort	Potentially lifetime, annual	CVD event free, non-fatal CHD, non-fatal CBVD, fatal CVD, fatal non-CVD, fatal all cause
11	Manyema et al. (2015) [42]	South Africa	-	Estimation of the effect of 20% SSB tax on the diabetes burden	Healthcare	Markov-multi state life table	Cohort	20 years, annual	BMI changes, diabetes
12	Wilcox et al. (2014) [43]	Syria	CHD IMPACT Model	Cost-effectiveness analysis of salt reduction policies	Public/private sector, healthcare	Cell-based model	Cohort	10 years, annual	CHD death
13	Collins et al. (2015) [44]	England	-	Projection of 20% of sugary drinks duty impact on disease events	Healthcare	Microsimulation	Individual	20 years, annual	Diabetes, stroke, CHD
14	Lawson et al. (2016) [45]	Scotland	Scottish CVD Policy Model	The development of model for conducting economic evaluation	N/A	Markov	Cohort	Potentially lifetime, annual	CVD event free, non-fatal CHD, non-fatal CBVD, fatal CVD, fatal non-CVD, fatal all cause
15	Sánchez-Romero et al. (2016) [46]	Mexico	CVD Policy Model-Mexico	Projection of SSB tax policies	N/A	Markov	Cohort	10 years, annual	No event, CVD event (MI, stroke, angina), death
16	Wang et al., (2016) [47]	China	CVD Policy Model-China	Estimation of the effect of population-wide salt restriction in China	Healthcare system payer’s	Markov	Cohort	Lifetime, annual	CVD free, acute CVD events, chronic CVD states, fatal CHD or stroke, non-CVD death
17	Breeze et al. (2017) [48]	UK	SPHR Model	Cost effectiveness analysis of different interventions for type 2 diabetes prevention	NHS/PSS	Microsimulation	Individual	Lifetime, annual	Metabolic profile, no diabetes, diabetes, complications, CVD, cancer, osteo, depression, mortality
18	Pandya et al. (2017) [49]	US	CVD- PREDICT	Description of the CVD model in detail; and performed model validation analyses	N/A	Microsimulation	Individual	Potentially lifetime	Disease free, CHD, stroke, death
19	Mozaffarian et al. (2018) [50]	US	CVD-PREDICT	Estimation of the health impact and cost-effectiveness in SNAP program	Societal and government	Microsimulation	Individual	5–20 years and lifetime, annual	No CVD, acute CHD, chronic CHD, repeat MI or CVA, acute CVA, chronic CVA, CVD or non-CVD death
20	Riveros et al. (2018) [51]	Brazil	Adaptation of Scottish CVD Policy Model	Calibration of Brazilian CVD model	N/A	Markov	Cohort	N/R	CVD event free, non-fatal CHD, non-fatal CBVD, fatal CVD, fatal non-CVD, fatal all cause
21	Schönbach et al. (2019) [52]	Germany	DYNAMO-HIA	Estimation of health impact of tax on processed meat	N/A	Markov (extend to microsimulation)	Individual	10 years, annual	Prevalence in CHD, diabetes, cancer
22	Huang et al. (2019) [53]	US	CHD IMPACT model	Estimation of the health impact and cost-effectiveness added sugar labelling on all packaged food and beverages	Healthcare and societal	Cell-based model	Cohort	20 years, annual	CHD incidence, stroke incidence, T2DM incidence
23	Salgado et al. (2019) [54]	Argentina	CVD Policy Model-Argentina	The update Argentina CVD Policy Model	N/A	Markov	Cohort	Lifetime, annual	CVD free, acute CVD events, chronic CVD states, fatal CHD or stroke, non-CVD death
24	Wilde et al. (2019) [55]	US	CVD-PREDICT	Estimation of the health impact and cost-effectiveness of a national penny per-ounce SSBs tax	Healthcare and societal	Microsimulation	Individual	Lifetime, annual	Disease free, CHD, stroke, death
25	Broeks et al. (2020) [56]	Netherlands	DYNAMO-HIA	Estimation of the effects of a tax on meat and a subsidy on FV consumption	Societal	Markov	Cohort	30 years, annual	Healthy, disease, death
26	Lee et al. (2020) [57]	US	CVD-PREDICT	Estimation of the health impact and cost-effectiveness of three SSBs tax designs	Healthcare, government, societal	Microsimulation	Individual	Lifetime, annual	Disease free, CHD, stroke, death
27	Liu et al. (2020) [58]	US	CVD-PREDICT	Estimation of the health impact and cost-effectiveness of federal restaurant menu calorie labelling policy	Healthcare and societal	Microsimulation	Individual	Lifetime, annual	Disease free, CHD, stroke, death
28	Salgado et al. (2020) [59]	Argentina	CVD Policy Model-Argentina	Estimation of the impact of reducing SSB consumption	N/A	Markov	Cohort	10 years, annual	CVD free, acute CVD events, chronic CVD states, fatal CHD or stroke, non-CVD death
29	Dehmer et al. (2020) [60]	US	Health Partners Institute’s Model Health	Evaluate prospective CVD related sodium reduction targets	Healthcare	Microsimulation	Individual	10 years, annual	Disease free, hypertension, CVD, post-CVD, death
30	Shangguan et al. (2021) [61]	US	CVD-PREDICT	Assessment of the effect of NSSRI (National Salt and Sugar Initiative) sugar reformulation policy	Healthcare and societal	Microsimulation	Individual	Lifetime, annual	Sugar intake, acute CVD, diabetes, chronic CVD, CVD or non-CVD death
31	Thomas et al. (2022) [62]	England	SPHR model	Estimation of health benefits, costs, and equity impact of food advertising across London transport network	NHS/PSS	Microsimulation	Cohort	Lifetime	Metabolic profile, no diabetes, diabetes, complications, CVD, cancer, osteo, depression, mortality
32	Lou et al. (2023) [63]	US	CVD Policy Model	Impact assessment of implementing SSB taxes and FV subsidies on long-term CVD outcomes and healthcare costs in NYC	Societal	Microsimulation	Individual	10 years, annual	Healthy, CHD, stroke, both CHD and stroke, CVD-related death, and non-CVD-related death

*CVD* Cardiovascular disease, *MI* Myocardial infarction, *CBVD* Cerebrovascular disease, *CHD* Coronary heart disease, *CVA* Cerebrovascular accident, *SSB* Sugar-sweetened beverage, *T2DM* Type 2 diabetes

### Description of included studies

From 32 articles retrieved, there is a diverse range of geographical study locations, including the US (*n* = 12) [34–36, 49, 50, 53, 55, 57, 58, 60, 61, 63], UK (*n* = 6) [39, 41, 44, 45, 48, 62], Netherlands (*n* = 1) [56], Germany (*n* = 1) [52], Latin America (*n* = 5) [37, 46, 51, 54, 59], South Africa (*n* = 1) [42], India (*n* = 1) [38], China (*n* = 3) [32, 33, 47], Eastern Mediterranean (*n* = 2) [40, 43].

Policy models were predominantly defined as computational simulations using mathematical frameworks to project population-level outcomes related to mortality, morbidity, disease burden, and economic costs. These models often quantified the associated costs and assessed the impact of policy interventions on health and economic outcomes. While some studies explicitly defined “policy model,” others implicitly employed this framework by using decision models for economic evaluation or health outcome projections evaluating interventions at a population level [36, 38, 39, 44].

All included policy models met the eligibility criteria by the capability to incorporating both epidemiological and economic parameters. However, the scope and depth of analysis varied across studies. Some models focused solely on clinical or health outcomes, such as CVD mortality or T2DM incidence, while others concentrated on cost and outcomes estimation or conducted full economic evaluations, such as cost-effectiveness analyses using metrics like the incremental cost-effectiveness ratio (ICER). The dietary policies examined were diverse, including interventions such as sugar taxes, salt reduction initiatives, and food labelling strategies. Notably, this systematic review prioritises the methodological aspects of model structure and application rather than the efficacy or effectiveness of specific public health interventions.

The systematic review identifies key features of several main policy models, each with its strengths, limitations, and potential applicability (Table 3). DYNAMO-HIA (Dynamic Modelling for Health Impact Assessment) is a model that quantifies policies’ impact on health determinants. It employs a Markov-based modelling approach, allowing for the simulation of a real-life population by explicitly considering risk factor states [52, 56]. DYNAMO-HIA focuses on assessing the health impacts of policies on non-communicable diseases (NCDs), including CVD and diabetes. Its strengths lie in its comprehensive analysis, though its complexity and substantial data requirements can pose implementation challenges [52, 56].

**Table 3:** Disease prevention policy models

	DYNAMO-HIA	CVD Policy Model	CHD Policy Model	Impact CHD	CVD-Predict	Scottish Policy model	SPHR Diabetes Model
Scope	NCDs (non-communicable diseases) including CVD, diabetes, and risk factors	CVD and related risk factors, focusing on prevention and treatment strategies	CVD and related risk factors, focusing on prevention and treatment strategies	CHD and CVD interventions, evaluating their effectiveness	CVD with a focus on prediction and risk stratification for better preventive measures	Public health with a specific focus on CVD and associated risk factors in Scotland	Diabetes and related risk factors, focusing on prevention, management, and health outcomes
Applicability	Primarily European countries, but adaptable globally	Primarily used in the US	Primarily used in the US	Applicable globally with regional adaptations	Applicable globally, with a focus on predictive analytics	Primarily used in Scotland	Primarily used in the UK
Data sources	European health surveys, epidemiological studies, and literature	National health surveys, clinical trials, epidemiological studies	National health surveys, clinical trials, epidemiological studies	National health surveys, clinical trials, epidemiological studies	National health surveys, clinical trials, epidemiological studies	Scottish health surveys, hospital records, national statistics	National health surveys, clinical trials, epidemiological studies
Outcome of interests	Estimates incidence, prevalence, mortality, QALY health impact, under various policy scenarios	Estimates incidence, prevalence, mortality, and healthcare costs, cost -effectiveness	Estimates incidence, prevalence, mortality, QALY, health disparities healthcare costs of CHD and stroke	Estimates incidence, mortality, hospital admissions, cost-effectiveness	Estimates incidence, risk prediction, mortality, and health care costs, health outcomes, cost -effectiveness	Estimates incidence, mortality, hospital admissions, QALE, cost-effectiveness	Estimates incidence, prevalence, mortality, QALY, cost-effectiveness
Key strengths	Comprehensive modelling of individual and population-level effects; integration of multiple risk factors and interventions for a nuanced analysis across health outcomes	Robust framework for evaluating interventions at a population level; flexible to include various types of interventions; extensive validation with US data	Extensive validation with US data; comprehensive risk factor integration	Comprehensive evaluation of interventions; focus on real-world applicability; extensive data sources	High granularity of individual risk prediction; ability to incorporate large datasets and update predictions with real-time data	Robust dataset specific to Scotland; focus on real-world applicability and policy impact; capable of addressing health inequalities and informing equitable policy decisions	Focus on diabetes-specific interventions and outcomes; ability to assess a wide range of potential interventions and their population-level impacts
Key weaknesses	Complexity in adapting to non-European contexts– Requires extensive data input	Can be complex to adapt to new populations or to integrate novel interventions without substantial effort and data	Requires extensive and high-quality data for accurate projections; complexity of model may limit its accessibility for non-specialists	May not account for all complex interactions between risk factors and interventions; data limitations can affect accuracy	Requires access to high-quality, comprehensive health records; model accuracy can be affected by missing or inaccurate data	Limited to the Scottish population, which may limit generalisability to other regions; data limitations outside of Scotland may affect model accuracy	Complexity and data requirements can limit accessibility for some users; relies on accurate input data for precise predictions

Meanwhile, the CVD Policy Model [37, 46, 59, 63], CHD Policy Model [32–35], and Scottish Policy Model [41, 45] evaluate CVD interventions at the population level using a state-transition model. These policy models are robust for evaluating population-level interventions but can be complex to adapt to new populations or interventions.

The SPHR (School of Public Health) Diabetes Model developed at the University of Sheffield is a predictive tool that calculates the risk of developing type 2 diabetes (T2D). It utilises a range of demographic, clinical, and lifestyle factors to generate personalised risk assessments, aiding in the prevention and management of diabetes [48, 62]. The SPHR Diabetes Model models the impact of diabetes prevention and intervention strategies at the population level using a system dynamics approach, with strengths in assessing diabetes-specific interventions but limitations due to complexity and data requirements [48].

In addition, the CVD-PREDICT (Cardiovascular Disease Policy Model for Risk, Events, Detection, Interventions, Costs, and Trends) also applied a microsimulation model to assess public health prevention programmes such as sugar-sweetened beverages (SSB) tax and consumption policies, or other dietary policies [49, 50, 55, 57, 58, 61]. The IMPACT study used a cell-based policy model, which is a sub-type of a compartmental spreadsheet-based microsimulation that generally provides aggregate estimates of population dynamics over time, in this case, the life years and mortality of coronary heart disease (CHD) or other non-communicable diseases [39, 40, 43].

Those models included common risk factors and baseline parameters such as age, sex, body mass index (BMI), systolic blood pressure (SBP), low-density lipoprotein (LDL)-cholesterol, high-density lipoprotein (HDL)-cholesterol, glycated haemoglobin (HbA1c), smoking and alcohol status, and other related factors. The structure of the model depends on the policy model itself, and most of them focus on a single disease (CVD or T2DM) model or assign a CVD/T2DM state as a risk factor or comorbid state.

Markov models have been the predominant approach in this review (47%) [32–35, 37, 41, 42, 46, 47, 52, 54, 56, 59] and microsimulations have been extensively performed in recent years (40%) [36, 38, 44, 48–50, 55, 57, 60–63]. Some studies also applied a simpler type of microsimulation model such as a cell-based model (13%) [39, 40, 43, 53]. Models are initiated with ‘disease-free’ or ‘healthy’ states followed by disease and death states, employing an annual cycle and a long-term horizon, (> 10 years or lifetime), allowing the quantification of health outcomes, benefits, and associated costs.

Overall, these models use various types such as microsimulation, state-transition, compartmental, and system dynamics to support their specific purposes and applications. They require information rich data sources like national health surveys and electronic health records for accurate predictions and assessments. While primarily used to inform policy decisions and guide public health strategies, these models vary in adaptability to different aims and health outcomes.

### Costs and outcomes

Costs incorporated in the models varied based on the policy questions and perspectives defined. Direct medical costs included expenses related to disease conditions, such as hospitalisation, healthcare provider services (consultations, treatments), medications, and laboratory/diagnostic procedures. Indirect costs were associated with productivity losses due to illness or disability, while programme costs referred to expenses incurred for implementing policy interventions. Approximately 72% of studies accounted for direct medical costs [33–35, 39, 40, 42–44, 46–48, 50, 52, 55–63], indirect costs were included in 38% of studies [39, 48, 50, 52, 55–61], depending on the analysis perspective. Programme costs were reported in 56% of studies, predominantly focusing on dietary interventions [35, 36, 38–40, 43, 44, 47, 48, 50, 52, 55–57, 62–65]. Monetary values were typically reported in USD or international dollars (Table 4).

**Table 4:** Costs and outcomes measured

Authors name	Costs			Outcomes				Discounting
	Direct healthcare costs	Indirect costs	Programme/implementation costs	Disease cases/ event	LY/LE	QALY/ DALY	ACER/ICER/ NMB	
Moran et al. (2008) [32]	√	-	-	√	-	√	-	√
Moran et al. (2010) [33]	N/R	N/R	N/R	√	-	-	-	N/R
Bibbins-Domingo et al. (2010) [34]	√	-	-	-	-	√	-	√
Wang et al. (2012) [35]	√	-	√	√	-	-	-	√
Basu et al. (2013) [36]	-	-	√	√	-	√	-	√
Konfino et al. (2013) [37]	N/R	N/R	N/R	√	-	-	-	N/R
Basu et al. (2014) [38]	-	-	√	√	-	-	-	N/R
Collins et al. (2014) [39]	√	√	√	√	√	-	-	√
Mason et al. (2014) [40]	√	-	√	√	√	-	-	√
Lewsey et al. (2015) [41]	N/R	N/R	N/R	√	√	-	-	N/R
Manyema et al. (2015) [42]	√	-	-	√	√	√	-	√
Wilcox et al. (2014) [43]	√	-	√	√	√	-	√	√
Collins et al. (2015) [44]	√	-	√	√	-	√	-	-
Lawson et al. (2016) [45]	N/R	N/R	N/R	√	√	-	-	√
Sánchez-Romero et al. (2016) [46]	√	-	√	√	-	-	-	N/R
Wang et al., (2016) [47]	√	-	√	√	-	√	-	√
Breeze et al. (2017) [48]	√	√	√	√	√	√	√	√
Pandya et al. (2017) [49]	N/R	N/R	N/R	√	-	-	-	N/R
Mozaffarian et al. (2018) [50]	√	√	√	-	-	√	√	√
Riveros et al. (2018) [51]	N/R	N/R	N/R	√	√	-	-	N/R
Schönbach et al. (2019) [52]	√	√	√	√	-	-	-	N/R
Huang et al. (2019) [53]	√	√	-	√	-	√	√	√
Salgado et al. (2019) [54]	N/R	N/R	N/R	√	-	-	-	N/R
Wilde et al. (2019) [55]	√	√	√	-	-	√	√	√
Broeks et al. (2020) [56]	√	√	√	√	-	-	-	√
Lee et al. (2020) [57]	√	√	√	√	-	√	-	√
Liu et al. (2020) [58]	√	√	√	√	-	√	√	√
Salgado et al. (2020) [59]	√	√	-	√	-	-	-	N/R
Dehmer et al. (2020) [60]	√	√	-	√	-	-	-	-
Shangguan et al. (2021) [61]	√	√	-	√	-	√	√	√
Thomas et al. (2022) [62]	√	-	√	√	-	√	√	√
Lou et al. (2023) [63]	√	-	√	√	-	√	√	√

*N/R* Not related, *LE* Life expectancy, *LY* Life year, *DALY* Disability-adjusted life year, *QALY* Quality-adjusted life year, *ACER* Average cost effectiveness ratio, *ICER* Incremental cost-effectiveness ratio, *NMB* Net monetary benefit

In terms of outcome measures, the majority studies (90%) estimated disease incidence or prevalence [32, 33, 35, 36, 39, 41, 43–49, 52–54, 56–63], while 47% reported generic health outcomes such as Quality-Adjusted Life Years (QALYs) or Disability-Adjusted Life Years (DALYs) [33, 34, 36, 42, 44, 47, 48, 50, 53, 55, 57, 58, 62]. While most studies estimated QALYs by assigning utility weights to different health states, the methodology for deriving these utility values is often poorly described. In many cases, utility values are sourced from previously published studies, but the papers do not provide detailed explanation of the methods used to derive these values-such as whether they were obtained through direct methods (e.g.: time trade-off, standard gamble) or indirect methods (e.g., EQ- 5D, SF- 6D) defines that estimating QALY by assigning the utility weights to different health states, this however poorly described in the paper, since most of them are derived from published studies. Hence, there are no detail information about the method of deriving utilities values (i.e.: direct or indirect methods).

Studies using decision models for full economic evaluations frequently reported incremental analysis metrics, such as the Incremental Cost-Effectiveness Ratio (ICER) and Incremental Net Benefit (INB) (28%) [43, 48, 50, 55, 58, 61, 62]. A small number of studies analysed how costs and benefits were distributed across demographic groups [41, 62].

Most studies incorporated discount rates for costs, outcomes, or both, ranging from 0 to 5%, with justifications based on local guidelines or applied discount rates in scenario and sensitivity analyses.

### Model validation

Model validation refers to the evaluation of whether a model accurately represents the system it seeks to simulate and whether its outputs provide a robust foundation for decision-making. Validation is essential to ensure reliability, accuracy, and credibility, thereby enhancing transparency, supporting evidence-based decision-making, and identifying potential limitations that require refinement [66, 67]. This review assessed five key types of model validation: 1) face validity, evaluates whether the model’s structure, inputs, and outputs logically reflect known behaviours and outcomes of specific diseases; 2) internal validity, assesses whether the algorithms and relationships within the model accurately simulate disease progression and interactions; 3) cross-validity, ensures that the model’s findings are consistent across different samples or populations within the same study; 4) external validity, examines the generalisability of the model to other settings, populations, or time periods; 5) predictive validity, tests the model’s ability to accurately predict real-world outcomes.

The findings of this review revealed that all studies conducted assessments of face and internal validity. Cross-validity was mentioned in one study [48]; however, the methodologies employed for testing were often unclear. External validation was performed in 53% of studies, indicating some efforts to evaluate the generalisability of models [32, 35, 39–41, 45, 47, 49, 50, 54, 55, 57–59]. None of the included articles reported predictive validation (Table 5).

**Table 5:** Validation test and uncertainty analysis

**Authors name**	**Validation test**						Uncertainty analysis		
	Face validity	Internal validity	Cross-validity	External validity	Predictive validity		Deterministic SA (DSA)		Probabilistic SA(PSA)
Moran et al. (2008) [32]	√	√	-	√	-		√		-
Moran et al. (2010) [33]	√	√	-	-	-		√		-
Bibbins-Domingo et al. (2010) [34]	√	√	-	Unclear	-		√		-
Wang et al. (2012) [35]	√	√	-	√	-		√		√
Basu et al. (2013) [36]	√	√	Unclear	-	√		√		√
Konfino et al. (2013) [37]	√	√	-	-	-		√		-
Basu et al. (2014) [38]	√	√	-	-	-		√		-
Collins et al. (2014) [39]	√	√	-	√	-		√		√
Mason et al. (2014) [40]	√	√	-	√	-		√		-
Lewsey et al. (2015) [41]	√	√	-	√	-		√		√
Manyema et al. (2015) [42]	√	√	-	Unclear	-		√		-
Wilcox et al. (2014) [43]	√	√	-	-	-		√		-
Collins et al. (2015) [44]	√	√	-	-	-		-		√
Lawson et al. (2016) [45]	√	√	-	√	-		√		√
Sánchez-Romero et al. (2016) [46]	√	√	Unclear	-	-		√		√
Wang et al., (2016) [47]	√	√	-	√	-		√		-
Breeze et al. (2017) [48]	√	√	√	-	-		√		√
Pandya et al. (2017) [49]	√	√	-	√	-		N/R		N/R
Mozaffarian et al. (2018) [50]	√	√	-	√	-		√		√
Riveros et al. (2018) [51]	√	√	-	√	-		-		-
Schönbach et al. (2019) [52]	√	√	-	-	-		-		√
Huang et al. (2019) [53]	√	√	-	√	Unclear		√		√
Salgado et al. (2019) [54]	√	√	-	√	-		N/R		N/R
Wilde et al. (2019) [55]	√	√	-	√	-		√		√
Broeks et al. (2020) [56]	√	√	-	-	-		√		√
Lee et al. (2020) [57]	√	√	-	√	-		√		√
Liu et al. (2020) [58]	√	√	-	√	-		√		√
Salgado et al. (2020) [59]	√	√	-	√	-		√		√
Dehmer et al. (2020) [60]	√	√	-	√	-		√		-
Shangguan et al. (2021) [61]	√	√	-	-	-		√		√
Thomas et al. (2022) [62]	√	√	-	-	-		√		√
Lou et al. (2023) [63]	√	√	-	-	-		√		-

### Model uncertainty and sensitivity analysis

Uncertainty is an inherent part of health economics and policy models. It arises from various sources and can significantly impact the results or conclusion of an analysis. Sensitivity analyses (SA) are commonly employed to explore these uncertainties, either deterministically or probabilistically [68]. Deterministic sensitivity analyses (DSA), such as one-way or scenario analyses, systematically examine the impact of uncertainty by incorporating plausible alternative values or scenarios. In contrast, probabilistic sensitivity analyses (PSA) assign probability distributions to uncertain parameters and perform multiple model simulations to produce a distribution of outcomes.

All studies included in this review reported conducting sensitivity analyses as part of their modelling process (Table 5). Of these, 50% (16 studies) performed both DSA and PSA, while the remainder employed only one type of sensitivity analysis [35, 36, 39, 41, 44–46, 46, 48, 50, 53, 55–57, 59, 61, 62].

### Quality appraisal

The quality of models was appraised using the Philips checklist [16], categorised into three distinct domains including structure, data, and consistency. The ‘structure’ domain assessed how well the model’s framework was constructed, including the clarity and appropriateness of the model’s design about the decision problem it aims to address. The ‘data’ domain evaluates the sources, quality, and appropriateness of the data used within the model. For internal and external consistency of the model, ensuring that the model’s outputs are logical and comparable with other models or data is part of the ‘consistency’ domain.

**Fig. 2:**
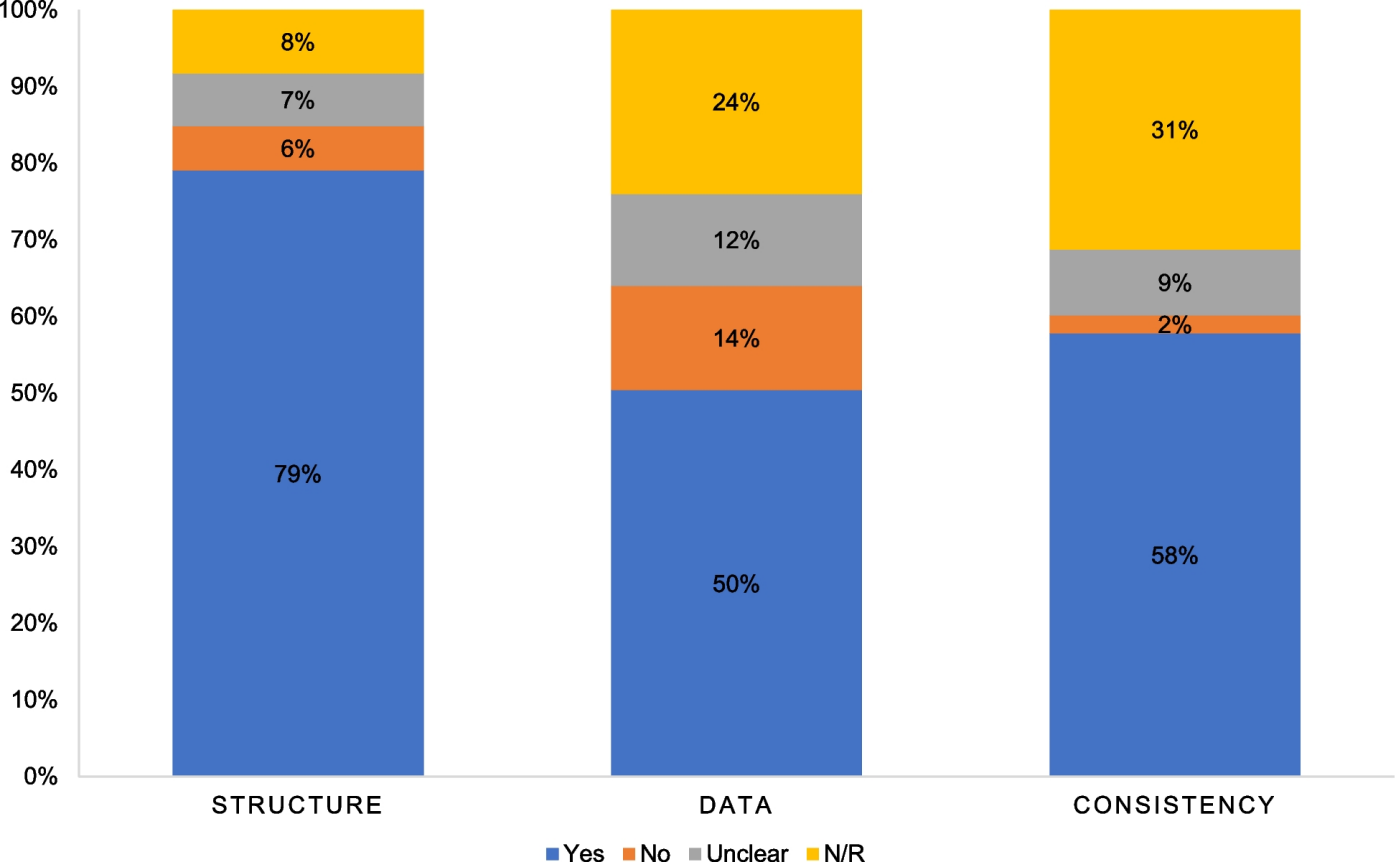
Summary of model’s quality

In Fig. 2, the blue colour represents “Yes” (indicating the criterion was fulfilled), orange represents “No” (indicating the criterion was not fulfilled), green indicates “Unclear” (where insufficient information or ambiguity was present), and light blue denotes “N/R” (not related or not applicable). The graph is based on cumulative percentages derived from each article’s responses.

Almost 80% of policy models met the criteria for the ‘model structure’ section. This category includes the appraisal of how the decision problem was constructed, encompassing the clarity of the decision problem, the study’s perspective, transparency, and consistency of model justification, input, and structural assumptions. Generally, model inputs and objectives were consistent with the stated perspectives and initial justifications. However, while the perspectives and settings were typically defined, not all models specified the decision-makers, despite the study results being intended for decision-maker use. Furthermore, most articles lacked explicit justification for the chosen time horizon and cycle length, although these were appropriately applied—likely due to the standard practice in modelling chronic diseases like CVD and T2DM. Also, the reasons for excluding certain options or alternative interventions were not always reported.

The cumulative quality of data and parameters used in the models was moderate (50%). This part of the appraisal focused on the data sources, the inclusion of parameters, and the methodological approaches reported in the articles. The models utilised a variety of data sources, including systematic reviews, meta-analyses, local and national epidemiological data, cost data, registries, administrative data, expert opinions, and other published sources. However, the quality assessment of the data incorporated into the models was often not clearly explained [35, 37, 39, 42, 59, 60]. A significant limitation was the lack of local representative data, which may have impacted final estimates and introduced high uncertainty into the results. To address this, many studies relied on data from other sources and constructed multiple assumptions [33, 35, 36, 38, 47].

Although face and internal validity seems subjectively well-reported, there was less clarity regarding the transparency of validation efforts, which may have been reported elsewhere or addressed implicitly without specifying the types of validation tests performed. Despite these gaps, most models did acknowledge aspects of consistency, particularly in model structure assumption and model parameter as well as defining outcomes of interests. All models provided clear evidence of internal assessment by conducting sensitivity analysis. The cross-validity and external validation were conducted such as by calibrating against independent data and reporting calibration results. The consistency of the articles was moderate to good (58%).

Overall, the review highlights a moderate to good quality across different aspects of the models, with notable strengths in model structure but areas for improvement in reporting data transparency and validation.

## Discussion

This systematic review offers a comprehensive critical appraisal of the methodological quality of the existing published CMD models. By evaluating the quality of these models, the findings provide valuable insights to inform and enhance the development process of a de novo policy model that can address some of the limitations identified and should be informed by a detailed conceptual model [69].

The review contributes to the existing evidence base by emphasising policy models capable of analysing prevention strategies for healthy or low-risk populations. This represents a departure from most previous policy models, which have predominantly focussed on summarising evidence for specific health interventions or technologies or are tailored to populations with moderate to high-risk profiles [15, 19, 20, 23, 25, 70–72]. Also, our review is the first study to consider non-single disease (T2DM and CVD) to represent the evidence related to CMDs.

A ‘policy model’ in this review is broadly defined to encompass various modelling approaches, including epidemiology-economic models, microsimulation models, and decision models, all of which contribute to informing health policy decision [33, 38, 41, 52, 58]. The distinction between policy models and decision models is often blurred, as decision models can be embedded within a broader policy modelling framework. For example, a policy model may incorporate decision-analytic components to answer specific questions—such as the cost effectiveness of an intervention—while simultaneously assessing its broader population-level and system-wide effects.

Given this overlap, this review adopts a comprehensive perspective, defining a policy model as framework designed to evaluate clinical/health outcomes, cost, cost-effectiveness, and broader societal implications of health interventions. This models play a crucial role in guiding public health policies and programs, aiming to reduce disease burden and improve population health by providing evidence-based projections of intervention impacts.

One of the clear advantages of modelling is the capability to estimate and simulate long-term disease progression and the impact of an intervention, which complements evidence generated in RCTs [73, 74]. Our review established that models were either simulated Markov-type cohort or individual-level models (microsimulation), with different perspectives chosen, costs incurred, and sensitivity analyses performed. Cohort simulations are advantageous for their efficiency and generalisability but are limited by their inability to account for individual variability, lack of precision, potential for ecological fallacy, and challenges in modelling complex interactions. In contrast, individual-level simulations offer greater granularity and personalised insights, capturing heterogeneity and specific outcomes, but they require extensive data, are resource-intensive, may involve significant uncertainty, and can be less interpretable and generalisable. The choice between these approaches depends on the study objectives, policy questions, and data availability.

Most policy models adopt a healthcare provider perspective; however, incorporating patient perspectives and accounting for potential productivity losses could provide a more comprehensive economic evaluation [75]. Given that the nature of CMD itself can significantly affect both patients'and caregivers'spending, a broader economic perspective may enhance policy relevance. However, existing studies reviewed do not provide further justification for not considering broader perspective, likely because the economic framework is typically established at the outset to align with specific policy questions.

The quality of models, as established in our appraisal does heavily rely on the quality of the data used. Many studies have highlighted concerns regarding the limited availability of representative or local data for model analysis. The lack of local clinical epidemiology data often necessitates the use of assumptions or non-local data, introducing uncertainty and raising concerns about data quality. While the use of published data from other sources can be valuable, issues regarding data transferability standards and the processes for adopting such data remain an issue. Justifications for data transferability were not consistently addressed in the reviewed studies, leading to reliance on multiple assumptions about parameters, which may introduce further limitations. Additionally, many models relied on survey and observational data (e.g., survey, self-reported non-local data), which is prone to under-reporting, selection bias, and recall bias, potentially affecting the accuracy of estimations.

The integration of real-world data (RWD) and updated local data holds substantial potential for improving model accuracy and representativeness. RWD refers to a health-related data that routinely collected outside of controlled clinical trials, such as electronic health records (EHRs), claims, billing data, registries, patient reported outcomes [76]. By capturing actual patient experiences and outcomes in routine clinical practice, RWD provides a more accurate reflection of diverse populations, enhancing the generalisability of findings and offering deeper insights into the real-world impact of healthcare interventions [77, 78]. However, the use of RWD presents its own challenges, including potential confounding variables, missing data, lead-time bias, and the inherent complexities of such datasets. Addressing these challenges effectively is critical to maximising the value of RWD in modelling analyses [79, 80]. From this review, CVD-PREDICT and Scottish Policy Model are arguably substantially leveraging RWD, using hospital records, large-scale health records, and national statistics which enhance the model’s validity and enables real-time updates for predictive modelling, ensuring their relevance in dynamic healthcare setting [41, 49].

Uncertainty is inherent in every modelling exercise, underscoring the need for improved reporting and characterisation of uncertainty. Additionally, it is crucial to report clear validity tests conducted to enhance the transparency of model development [81]. The model validation process was mostly not extensively discussed in published articles or overlapped terms in validation itself in publication-related health economic studies, thus limiting the reporting quality.

Addressing equity considerations in health economic analysis can enhance overall impact of policy decisions [82, 83]. Policies designed solely on cost-effectiveness without considering equity can lead to interventions that are efficient at an aggregate level but exacerbate existing inequalities. By integrating equity, policymakers can design more holistic interventions that balance efficiency with fairness, leading to more socially acceptable and sustainable health policies.

Several models reviewed in this study have incorporated equity analysis. The study by Thomas et al. [62] highlights that individuals from the most socioeconomically deprived group experience greater gains in QALYs compared to those from the least deprived groups. Additionally, within three years post-intervention, the policy was estimated to significantly reduce the cardiometabolic disease incidence in disadvantages communities, thereby contributing to equity improvement. Similarly, model developed by Lewsey et al. [41], emphasise the importance of equity assessment by analysing how different socioeconomic groups are affected by policies, helping to identify strategies that reduce health inequalities.

The overall quality of the models in this review is good. Most of the important model features are well-reported. However, in line with several current systematic literature reviews [65, 84, 85], not all policy models are fully comparable, due to the different model assumptions, modelling approaches, perspectives, and outcomes generated from the model.

We acknowledge several potential limitations in this review. First, this review only focused on articles reporting on very specific dietary policy interventions. We aimed to focus on critically appraising the model used, rather than assessing the health-economic result of any intervention. There are probably many primordial public health strategies besides dietary interventions, such as physical activities or smoking cessation policies. Second, the various applications of the policy model objectives and input parameters may affect the generalisability of findings from this review. Variability in assumptions, data sources, and methodological choices across models can influence conclusions, highlighting the need for careful interpretation when applying results to different contexts. Third, data extraction was conducted by a single reviewer, while 20% of studies were independently verified by independent reviewers, with discrepancies resolved through team discussions. However, this approach remain aligns with standard systematic review practices, ensuring consistency and minimising potential bias [28, 31].

This review focuses on policy models explicitly designed for population-level dietary interventions in the prevention of CMDs. However, it is important to acknowledge that some decision models originally developed for clinical interventions (e.g., pharmacological treatments for CVD and T2DM) may also be adaptable for evaluating dietary policies. While these models were built to assess individual level treatments, their structure—such as modelling disease progression and risk factors—could allow them to be modified for population-level interventions.

By excluding these general-purpose models, some potentially adaptable frameworks were not assessed in this review. Future research should explore whether clinical decision models can be extended for dietary policy evaluation, bridging the gap between individual treatment decision and population-level policy assessments.

Based on this systematic review, we propose the following recommendations to enhance the development of CMD policy models (Table 6):

**Table 6:** Evidence-based recommendations for CMD policy modelling

Area	Key recommendations
Model selection	State-transition models (e.g., Markov models) are commonly used for CMD progression, but analysts should align the model choice with the policy question, available data, and computational feasibility
Integration of CMDs	Given the shared risk factors of T2DM and CVD, incorporating them into the same model can improve accuracy and capture event-related risks
Risk factors	Models can integrate modifiable risk factors (e.g., BMI, cholesterol, lifestyle changes) to ensure more realistic projections
Data quality	High-quality patient-level and representative epidemiological data should be prioritised. Incorporating clinical biomarkers and capturing heterogeneous effects can improve generalisability
Economic perspective	If data are available, considering a societal perspective can enhance health-economic modelling beyond direct healthcare costs
Uncertainty analysis	Specifying uncertainty and conducting appropriate sensitivity analyses is essential for ensuring robust conclusions
Validation	Reporting validation tests (internal, external, face validity) is recommended to improve model reliability and reproducibility
Transparency & reporting	Clearly document model rationale, assumptions, and methodologies. Conceptual models should be well-documented to enhance credibility
Equity & distributional analysis	Ensuring that models assess distributional impacts can support policies that reduce health inequalities
Reproducibility & open Science	Adhering to best research practices and making policy models open source can improve transparency, accessibility, and reproducibility

## Conclusions and recommendations

In conclusion, the policy models reviewed herein show promising insights for informing policy decisions, particularly in the context of public health prevention strategies. Based on this systematic review, several recommendations are established to enhance the development of CMD policy models.

## Supplementary Information

Supplementary Material 1.

Supplementary Material 2.

## Data Availability

All data generated or analysed during this study are included in this published article and its supplementary material.

## Abbreviations

CMD: Cardiometabolic disease
CVD: Cardiovascular disease
DALY: Disability adjusted life years
DSA: Deterministic sensitivity analysis
ICER: Incremental cost effectiveness ratio
LY: Life years
NMB: Net-monetary benefit
PSA: Probabilistic sensitivity analysis
QALY: Quality adjusted life years
QALE: Quality adjusted life expectancy
SSB: Sugar-sweetened beverage
T2DM: Type 2 diabetes mellitus
