# Supplementary material for: Policy models for preventative interventions in cardiometabolic diseases: a systematic review
Source: BMC Health Serv Res. 2025 May 2;25:635. doi: 10.1186/s12913-025-12781-y (PMC12046856; doi:10.1186/s12913-025-12781-y)
Supplement: Supplementary file 1 — Supplementary Material 1. [file 12913_2025_12781_MOESM1_ESM.docx]

**Additional information 1. Searching Strategy**

**EMBASE (OVID)**

1 exp Diabetes Mellitus/

2 exp Diabetes Mellitus, Type 2/

3 (type* adj1 ("2" or "II" or two*) adj2 (diabete* or diabeti*)).mp.

4 (T2D or T2DM).mp.

5 exp Dyslipidemias/

6 exp Insulin Resistance/

7 exp Glucose Intolerance/

8 1 or 2 or 3 or 4 or 5 or 6 or 7

9 exp Cardiovascular Diseases/

10 (CV or CVD).mp.

11 exp Stroke/

12 exp Hypertension/

13 exp Myocardial Infarction/

14 (cardiovascular disease* or heart disease* or ischaemic heart disease* or ischemic heart disease* or angina or coronary disease* or cardiac or vascular disease* or cerebrovascular or cerebral vascular).mp.

15 9 or 10 or 11 or 12 or 13 or 14

16 exp Metabolic Diseases/

17 (metabolic adj1 (disease* or syndrom* or dysfunction* or disorder*)).mp.

18 (cardiometabolic or cardio-metabolic).mp.

19 16 or 17 or 18

20 8 or 15 or 19

21 (model* adj2 (decision* or analys* or simulat* or predict* or statistic* or mathematic* or state transition or Markov or discrete event simulation*)).mp.

22 20 and 21

23 exp Health Policy/ or health polic*.mp.

24 public health policy.mp.

25 (policy model* or health policy model* or diabetes polic* or cardiovascular polic* or cardiometabolic polic*).

26 (policy adj2 (disease* or epidemiolog*)).mp.

27 23 or 24 or 25 or 26

28 22 and 27

29 limit 28 to (english language and yr="2000 - 2022")

**Similar terms to MEDLINE, CINAHL, Google Scholar, OpenGrey*
